# Service Providers’ Attitudes Toward Mandatory Reporting of Intimate Partner Violence: The Impact of Professional Experience

**DOI:** 10.1177/08862605241305145

**Published:** 2024-12-27

**Authors:** Christine Nordby, Kevin S. Douglas, Solveig Karin Bø Vatnar

**Affiliations:** 1 Molde University College, Molde, Norway; 2 Simon Fraser University, Burnaby, BC, Canada; 3 Centre for Research and Education in Forensic Psychiatry, Bergen, Norway; 4 Centre for Research and Education in Forensic Psychiatry Oslo University Hospital, Oslo, Norway

**Keywords:** disclosure of domestic violence, domestic violence, legal intervention

## Abstract

Mandatory reporting (MR) among service providers (SP) working with intimate partner violence (IPV) is controversial, and the research is scarce. The potential association of SPs experience with IPV and MR-IPV and their attitudes is the aim of the current study. A total of 374 SPs working with victims and perpetrators (help-seekers) of IPV participated in this study. Factor analysis was conducted to produce a continuous standardized dependent variable as a measure of attitudes toward MR-IPV. Descriptive results indicated that SPs were generally supportive of MR-IPV. Linear regression analyses showed that a higher degree of professional experience with IPV was significantly associated with less skepticism of MR-IPV. The result was significant regardless of (1) severity of violence, (2) category of help-seeker, (3) time of incidents, and (4) number of cases where MR-IPV was considered relevant. Having substantial experience with IPV and MR-IPV remained significant adjusting for the SP category, years in current position, knowledge of MR, and frequency of mandatory reports. Our results indicate that experience is an important part of SPs attitudes toward MR-IPV.

## Introduction

Intimate partner violence (IPV) has been established as a serious world health issue (World Health Organization [WHO], 2012) and particularly a serious threat to women’s safety and lives (e.g., Stöckl et al., 2013; United Nations Office on Drugs and Crime, 2022). One action to prevent IPV and intimate partner *homicide* (IPH) is the law of mandatory reporting (MR). Disclosure *required* by law is commonly referred to as MR, while disclosure *allowed* by law is commonly referred to as discretionary reporting. Although laws vary among countries and states, the essence of MR is the same—by law certain individuals or groups are mandated to report certain serious crimes (and/or their impending occurrence) to the authorities. The purpose of mandatory reporting of IPV (MR-IPV) is to protect persons subjected to IPV from the risk of harm, to relieve them of having to report abusive partners themselves, and to improve the involvement of law enforcement and the criminal justice response (Jordan & Pritchard, 2018; Rodriguez et al., 2002; Vatnar et al., 2017). Furthermore, MR-IPV sends a signal to abusive partners that IPV is a serious crime (WHO, 2013) and establishes third-party documentation that may be critical to a criminal or civil legal action (Antle et al., 2010).

Many countries practice a form of mandatory reporting (e.g., *Australia*: Mathews & Walsh, 2014; *Cypros*: Panayiotopoulos, 2011; *Netherlands*: Van Dam et al., 2015; *United States of America* (*US*): Lund, 1999; McDowell et al., 1994). However, who is obligated to report what must be reported and to whom a report is made varies between countries and states. For instance, in Kentucky, all individuals are mandated to report to the Cabinet for Human Resources if they suspect abuse, neglect, or exploitation (Mandated Reporter Training, 2022). In California, only health practitioners are mandated reporters and must report to the police if they treat a physical injury they suspect was caused by a firearm or assaultive or abusive conduct (Mandated Reporter Training, 2022). In contrast, five states in the United States (Alabama, Louisiana, South Carolina, Washington, and Wyoming) do not have mandatory reporting laws for domestic violence (Mandated Reporter Training, 2022). Furthermore, MR-IPV is controversial (e.g., Lippy et al., 2019; Rodriguez et al., 2002). At the root of this discourse is the moral balance between (1) the state’s obligation to protect and chase justice on behalf of victims of IPV *and* (2) the victim’s autonomy over their own life and family. Historically, this is reflected in the growth of women’s rights and awareness of “battered women” in the 1960s and 70s, and calls for public intervention (Barner & Carney, 2011).

As mentioned above, one means of intervention is mandatory reporting, which in many jurisdictions simply means a report must be made to law enforcement about patient injuries. However, criticism has surrounded MR-IPV in contexts in which children might be removed from the home, and the perpetrator could punish the victim (Rodriguez et al., 1998). It is not illegal to stay in an abusive relationship, and victim advocates argue victims should be allowed to choose not to have the abuse reported if other consequences are more feared (Walker, 2017). On the other hand, there is a lack of evidence that suggests victims do not want it reported (Vatnar et al., 2021). Victims might also be in a difficult position to report on their own, for instance, because they fear the consequences or feel the violence is not severe enough (Del Rio & Del Valle, 2016; Robinson et al., 2020).

The current recommendation from the WHO (2013, 2017) is to abolish the implementation of MR-IPV, arguing that it might deter victims from seeking help. The recommendation is based on literature suggesting that service providers (SPs), that is, any professional who works with or meets a victim or perpetrator, are generally negative to MR because of this possible deterrent effect. However, a recent systematic review revealed that the MR-IPV research is limited and has serious methodological issues (Vatnar et al., 2021), and hence further clarification of SPs’ attitudes toward MR is necessary.

### Attitudes Toward MR-IPV

Vatnar et al.’s (2021) systematic review revealed indications of skepticism toward MR-IPV among SPs. However, the authors of the review found that methodological bias was high amongst the articles included in the review (i.e., systematically reporting findings of statistical minorities who opposed MR as being more significant than those of statistical majorities who supported MR-IPV; items asking if help seekers were “*warned* about MR”). The authors concluded that there was a gap between SPs having (1) strong attitudes toward MR-IPV, but (2) limited awareness of the law of MR-IPV (e.g. Cho et al., 2015; Sawyer et al., 2014), and (3) scarce experience with MR-IPV (Smith et al., 2008). Accordingly, the field requires further investigation.

The studies that aimed at exploring SPs’ low compliance with MR-IPV mainly measured barriers to reporting (i.e., circumstances inhibiting reporting) rather than attitudes *per se*. One study explored perceived barriers to reporting among 181 nurses in Florida (Smith et al., 2008). Of 16 potential barriers, the top 3 were: not enough evidence (32%), patient objected (17%), and confidentiality (9%). However, most attention was given to the nurses’ own personal experiences with being an IPV victim, which significantly influenced reporting behavior (IPV victims reported more often). Barriers were not explored further. Mgopa et al. (2021), through focus groups of health personnel and students in Tanzania, reported that participants mentioned several barriers that made managing IPV cases difficult, including a lack of knowledge about MR-IPV criteria. Health personnel were also more positive about reporting to the police than students were. Accordingly, the current study explored attitudes, not just barriers, toward MR-IPV and how actual professional experience with IPV and MR might be associated with attitudes.

Only two, though older, studies analyzing attitudes beyond a descriptive level were covered by Vatnar et al.’s (2021) systematic review. First, a study conducted in California measured approximately 500 physicians’ experiences with IPV and their attitudes toward MR-IPV (Rodriguez et al., 1999). More than half of the participants (59%) responded that they would not report (despite MR) if their patient objected. Participants who were unaware (self-reported) of the MR law had triple the likelihood of not reporting IPV if their patient objected. Descriptively, participants agreed with both supporting and opposing statements of MR. However, the majority (90%) agreed that under certain conditions (e.g., pregnancy, access to guns) they should report. Group differences were observed between medical specialties (e.g., family medicine vs. gynecology vs. emergency). Items measuring professional experiences with IPV and attitudes toward MR-IPV were not explored in any inferential statistical analyses. In addition to comparing differences between medical specialties, there are other professional groups who are confronted with decisions about MR-IPV. As such, the current study included a variety of different professions managing IPV.

Second, Tilden et al. (1994) questioned 1,521 different health professionals (i.e., dental hygienists; dentists; nurses; physicians; psychologists; social workers) about their attitudes toward abuse intervention and their management of it. The participants were asked about their attitudes toward MR regarding elder, spousal, and child abuse. Overall, participants, regardless of profession, considered MR for spousal abuse more effective (*M* = 63%, range 55%–76%) than for child (*M* = 43%, range 36%–52%) and elder abuse (*M* = 35%, range 32%–39%). The fact that two of three of the most relevant articles were published decades ago also underscores the need for further research on this topic.

### Theoretical Framework

Classic studies have illustrated that behavior is determined by many factors other than attitudes and that these affect attitude-behavior consistency. Most theories concerning attitude and behavior aim at understanding how attitudes predict behavior (Forgas et al., 2010; Haddock & Maio, 2004), not how experiences might influence attitudes. The former is what might be called behavior-change focused, and the latter attitude-change focused. Cognitive dissonance (Festinger, 1957) and attitudinal ambivalence (Harreveld et al., 2015) are theories that might contribute to explaining how experiences shape attitudes. The former might explain how past behaviors increase an internal cognitive conflict for individuals when faced with experiences that go against their own attitudes. The latter suggests that there are some topics individuals feel stronger toward than others and that it is possible to hold both positive and negative attitudes toward a target at one time. Hence, it might be that experiences only modify a person’s attitude toward someone or something rather than change it completely. As such, the theories might explain the possible outcomes of change in attitudes in face of attitude-contradicting experiences. In the context of MR-IPV, this could imply that SPs might have previous experiences with reporting under MR-IPV which could influence their evaluation of utilizing MR-IPV in the future, either negatively or positively, in relevant situations with a victim or a perpetrator of IPV. The current study, though not a formal test of theories of attitudes, is informed by these theoretical frameworks.

### Context of the Current Study

This research was conducted in Norway, where the main purpose of MR is to avert or prevent current or future crime, or the consequences thereof. Thus, the duty only applies when prevention is in fact possible. This law, as a means of preventing harm, is regulated in section 196 of the penal code (Straffeloven, 2005). Section 196 refers to 52 criminal acts that are covered by MR. Among them are domestic violence (sections 282 and 283). In this jurisdiction, MR applies to all individuals and is required even in cases where the information is initially confidential. The duty to report may be executed by notifying the police or by averting the criminal act or its consequences “by other means.” Failure to report according to section 196 is a punishable crime, unless reporting would expose the reporter, their next-of-kin, or an innocent person, cause a charge or indictment, or cause risk to life or welfare.

### Aim and Research Questions

The overall aim of the present study was to investigate attitudes toward MR-IPV among professionals working with IPV. We examined the following research questions:

1. What are the descriptive professional experiences with and attitudes toward MR-IPV among SPs?
2. To what extent are there associations between attitudes toward MR-IPV and professional experiences with MR-IPV, and IPV itself?
3. Are there group differences amongst SP professions or their sociodemographic characteristics regarding attitudes toward MR-IPV?

## Methods

The current study is a quantitative part of a larger project called MANREPORT-IPV. This project examines awareness, attitudes, and professional experiences with MR-IPV among IPV victims and perpetrators and SPs who work with IPV in Norway. The study was approved by Oslo University Hospital’s Data Protection Official reference number: 22/00221. Regional Committees for Medical Research Ethics (REK) deemed the study to be health service research, not health research, and hence not within their mandate (Reference number: 257644).

### Procedure

Data were collected between March 2022 and January 2023. Recruitment was carried out through planned annual meetings arranged by the SPs’ institutions (i.e., police; IPV treatment centers; domestic violence shelters) where the MANREPORT-IPV research group was allocated time (usually 15 min) to present the project (either online or in-person) for potential participants or their managers. Altogether, approximately 15 meetings were held, online or in person, to recruit participants. During online meetings, potential participants were encouraged to contact the first author to receive a link to the online questionnaire. In person, participants were given a questionnaire and consent form, and time was allocated to complete both. Most participants completed the materials in 45 to 50 min were used to complete the questionnaire, although with some variation. The researchers informed prospective participants that participation was voluntary, and they were given contact information to researchers in case they wished to withdraw. Their identity was concealed by cross-referencing their contact information on their consent form with a unique number in a separate file. Online, participants were given a link to identical online study materials and a randomly generated four-digit ID code, which they could use in case they wished to withdraw from the study.

### Measures

A questionnaire was developed comprising measures for attitudes, awareness and professional experience (adjusted for measuring MR-IPV), as well as new items created specifically for the current project. We review the measures used for the current analyses.

#### Attitudes Regarding IPV

The study adjusted items from the validated questionnaire used by the (Norwegian) Institute for Studies of the Medical Profession (Gaasø et al., 2019). The original questionnaire has used repeated measurements on 2,200 doctors every other year since 1992. The content covers Likert-scale statements on attitudes, awareness, and professional experiences, on societal and organizational conditions for professional work, ethics, values, and prioritization (see Table 1 for the adjusted statements.) We also included gender (options: male, female, or other), age, county of workplace and home, years in current position, and profession.

**Table 1.** Results of Items Regarding General Attitudes to Mandatory Reporting of Intimate Partner Violence Reported in *n* and Valid Percentages.

Item	Completely Disagree, *n* (%)	Somewhat Disagree, *n* (%)	Somewhat Agree, *n* (%)	Completely Agree, *n* (%)	I Don’t Know/Not Applicable, *n* (%)	Total, *N*
The duty to avert severe violence, that may lead to severe harm or death, must take precedence over client confidentiality	8 (2.2)	2 (0.6)	11 (3.1)	334 (92.8)	5 (1.4)	360
Mandatory reporting is important to prevent severe abuse and/or intimate partner homicide	5 (1.4)	1 (0.3)	25 (7)	322 (89.9)	5 (1.4)	358
Balancing client confidentiality and mandatory reporting is part of the professional practice	19 (5.2)	13 (3.6)	53 (14.6)	269 (74.1)	9 (2.5)	363
The welfare of children is more important than the welfare of the patient/client/consumer	28 (7.7)	13 (3.6)	54 (14.8)	260 (71.4)	9 (2.5)	364
Mandatory reporting is important to ensure that victims of intimate partner violence do not have to report the risk of severe abuse and/or of intimate partner homicide themselves	10 (2.8)	14 (3.9)	91 (25.5)	233 (65.3)	9 (2.5)	357
I generally comply with the mandatory reporting requirements when treating patients/clients/consumers	4 (1.1)	12 (3.4)	119 (34.1)	172 (49.3)	42 (12)	349
I find other ways to cooperate with, for example, the police, without using mandatory reporting	54 (15.3)	68 (19.3)	146 (41.5)	12 (3.4)	72 (20.5)	352
The distinction between the Health Personnel Act and mandatory reporting is unclear	35 (10.4)	62 (18.5)	106 (31.5)	16 (4.8)	117 (34.8)	336
I consult the legislation when unsure if mandatory reporting applies in patient/client/user treatment	16 (4.6)	32 (9.1)	102 (29.1)	157 (44.9)	43 (12.3)	350
Unless the patient poses a significant risk to others, the help services should not overrule the wishes and decisions of adults	85 (24.1)	116 (33)	112 (31.8)	21 (6)	18 (5.1)	352
Everyone has the right to a physician/therapist/healthcare provider/contact person with absolute client confidentiality?	118 (32.4)	80 (22)	105 (28.8)	50 (13.7)	11 (3)	364
If I disclose confidential information, patients/clients/consumers will lose trust in me, regardless of justification	53 (14.6)	145 (39.8)	120 (33)	24 (6.6)	22 (6)	364
The patient’s wish matters most; if they don’t want to report to the police, I won’t do it	166 (45.9)	123 (34)	53 (14.6)	10 (2.8)	10 (2.8)	362
If I disclose confidential information, I will feel unprofessional, regardless of justification	187 (51.4)	103 (28.3)	41 (11.3)	24 (6.6)	9 (2.5)	364
I find that my professional autonomy is reduced due to mandatory reporting	187 (54)	84 (24.3)	22 (6.4)	4 (1.2)	49 (14.2)	346
It only gets worse if you involve the police	208 (58.3)	116 (32.5)	19 (5.3)	0	14 (3.9)	357
Adults know best what they need, and should not be covered by the mandatory reporting law	256 (71.3)	85 (23.7)	13 (3.6)	1 (0.3)	4 (1.1)	359

*Note.* Total sample size = 374.

#### Professional Experience with IPV and MR-IPV

We developed questions about work experience with IPV and MR-IPV (number of cases with victims and perpetrators). We measured this in intervals of 10 cases (i.e., 1–5, 6–10, 11–20, 21–30, etc.) from none to more than 100 cases. Concerning professional experience with IPV, participants were asked to include any form thereof (i.e., physical, psychological, or sexual). Severe IPV was defined according to the Norwegian penal code for domestic violence and the Norwegian translation (Bendixen, 2005) of the revised Conflict Tactics Scale (CTS2) (Straus et al., 1996). The definitions were described in the questionnaire before the relevant questions as behavior that caused or could cause serious injury or death; was persistent abuse; and/or involved the use of a knife or other dangerous tool/weapon. Additionally, participants were asked if they had professional experience with victims of severe physical injury and perpetrators who have caused severe physical injury.

### Participants

Participants (*N* = 374) included individuals working with IPV victims and perpetrators in six different agencies: police (*n* = 42); child welfare services (*n* = 42); emergency primary health care (emergency rooms and assault centers) (*n* = 83); anger management (*n* = 67); domestic violence shelters (*n* = 98); alternative to violence (*n* = 42). Both the emergency room and assault centers are part of the Norwegian emergency primary health care and work in tandem. SPs very often work shifts in both the emergency room and assault centers and never work completely independently; hence, these groups have been combined. Alternative to violence and anger management both offer therapy to individuals who struggle with issues of violence perpetration. However, they are two independent agencies, in addition to alternative to violence offering therapy to victims of violence. As such these agencies are separated into two groups.

The mean age of participants was 45 (*SD* = 10), and they had worked, on average, 10 years (*SD* = 8) in their current position. Most participants were female (79%), 21% were male, and no participants identified themselves as “other gender.”

All participants had some experience working with victims or perpetrators of IPV or both categories and in various degrees of severity. Over half (52%) of participants had experienced between 1 and 50 cases with victims of IPV (no cases = 0.5%). About half (51%) of participants had experienced 1 to 20 cases of victims of *severe* IPV (no cases = 8.3%). Half (50%) of participants had experienced 1 to 10 cases of victims of *severe physical injury* (no cases = 15.8%). More than half (55%) of participants had experienced 1 to 30 cases with perpetrators (no cases = 10.4%) and 1 to 20 cases with perpetrators of *severe* IPV (55%) (no cases = 21.9%), and 56% of participants had experiences with 1 to 20 cases with perpetrators of *severe physical injury* (no cases = 30.7%). Missing for variables within this section = <2%.

Four percent of participants had professional experience with both incidents where a victim was murdered by an intimate partner and a perpetrator had committed IPH. Five percent had only experience with a victim of IPH, while 4% had only experience with a perpetrator of IPH. The number of cases for those who had these experiences ranged from 1 to 6 cases for victims of IPH, and 1 to 10 for perpetrators of IPH.

### Analyses

Basic descriptive analyses were used (frequencies; means; standard deviations) to investigate SPs’ attitudes toward MR at the item level. All variables had some missing values (range 3–81; *M* = 32 missing values; *SD* = 16), which were deleted pairwise in analyses. Uni- and multivariate linear regression were utilized to test if independent variables were associated with attitudes toward MR-IPV. STATA version 16.1 (StataCorp LLC) was used to conduct factor analyses (see section below); IBM SPSS version 29 was used for all other analyses. Multicollinearity was not present for any relevant independent variables (variance inflation factor <10).

#### Factor Analyses: Attitudes Toward MR-IPV

All items regarding perceptions of and attitudes toward MR-IPV were added to an exploratory factor analysis (see Table 2) to investigate if there was a factor solution that captured attitudes toward MR-IPV. The analysis produced three factors with eigenvalues greater than 1. Due to the exploratory nature of this study, the factor loading threshold was set to 0.30 (Costello & Osborne, 2005; Fabrigar et al., 1999; Tabachnick & Fidell, 2001). Four items did not have a factor loading above 0.3, and therefore were excluded. Item 2 was moved from one factor to another for theoretical reasons. When deciding which factors would most adequately measure attitudes toward MR-IPV on a scale, factor 2 was deemed appropriate. Factor 1 was considered to cover professional experience with practice and concerned items on the perception of the use of MR-IPV among colleagues, leaders, and other agencies. Factor 2 covered items on evaluating confidentiality (e.g., If I disclose confidential information, patients/clients/users will lose trust in me, regardless of justification), and perception of autonomy of the patient/client/user (e.g., The patient’s wish matters most; if they don’t want to report to the police, I won’t do it.) Factor 3 did not have a clearly interpretable theme. All items used in the exploratory factor analysis were then added to a confirmatory factor analysis, which produced a root mean square error of approximation (RMSEA) of 0.065, and a comparative fit index (CFI) of 0.867. Participants who responded “I don’t know” to any item were removed from scale construction, which reduced the *N* for these analyses from 374 to 265. The final attitude score (“Skepticism to MR-IPV”) was standardized (*M* = 0, *SD* = 1), and the items included in this factor produced an alpha of .694.

**Table 2.** Exploratory Factor Analysis with Oblique Rotation for Items Regarding Attitudes to MR-IPV.

Item	Factor 1	Factor 2	Factor 3
Everyone has the right to a physician/therapist/healthcare provider/contact person with absolute client confidentiality?		**0.5053**	
If I disclose confidential information, patients/clients/users will lose trust in me, regardless of justification		**0.5498**	
If I disclose confidential information, I will feel unprofessional, regardless of justification		**0.5791**	
Balancing client confidentiality and mandatory reporting is part of the professional practice			
The patient’s wish matters most; if they don’t want to report to the police, I won’t do it		**0.4566**	0.3074
The welfare of children is more important than the welfare of the patient/client/user			
I find other ways to cooperate with, for example, the police, without using mandatory reporting			**0.5627**
The duty to avert severe violence, that may lead to severe harm or death, must take precedence over client confidentiality			
Unless the patient poses a significant risk to others, the help services should not overrule the wishes and decisions of adults			**0.3619**
Adults know best what they need, and should not be covered by the mandatory reporting law		0.3570	**0.3781**
It only gets worse if you involve the police			**0.4238**
Mandatory reporting is important so that victims of violence can avoid making a report themselves or warning them about the risk of severe abuse and/or IPH			
Mandatory reporting is important to prevent severe abuse and/or intimate partner homicide			
I generally comply with the mandatory reporting requirements when treating patients/clients/users	0.4011	**−0.4198**	
I consult the legislation when unsure if mandatory reporting applies in patient/client/user treatment			
The distinction between the Health Personnel Act and mandatory reporting is unclear			
I find that my professional autonomy is reduced due to mandatory reporting		**0.3418**	
Are you informed about the criteria that should be used to make a decision regarding the application of mandatory reporting within your field?		**−0.3580**	
If life and health are at stake, is it your impression that mandatory reporting is usually complied with?	**0.5841**		
If life and health are at stake, is it your impression that mandatory reporting is usually complied with by your leaders?	**0.7929**		
If life and health are at stake, is it your impression that mandatory reporting is usually complied with by your colleagues?	**0.8006**		
If life and health are at stake, is it your impression that mandatory reporting is usually complied with by other help services?	**0.5187**		

*Note.* Eigenvalue factor 1 = 2.36213; Eigenvalue factor 2 = 2.09610; Eigenvalue factor 3 = 1.06057. *N* = 265. MR-IPV = mandatory reporting of intimate partner violence; IPH = intimate partner *homicide*.

## Results

### Descriptives

#### Professional Experience with MR-IPV

With 10 case intervals from 1 to above 100, we measured SPs frequencies of cases where MR was considered relevant (see Table 3). Over half of the participants responded that they had never experienced MR-IPV being relevant when working with a perpetrator while just under a third responded the same regarding victims. Of those who had experience with MR-IPV being relevant regarding perpetrators about a quarter found it relevant for one to five cases, while the remaining 15% of participants responded anywhere between six cases and above 100 (see Table 3). Regarding the victims, just above a third found it relevant for one to five cases, while the remaining 31% responded anywhere between six cases to above 100 (see Table 3). The large majority of participants had never chosen not to report because a victim or perpetrator told them not to, both within the last 12 months and throughout their careers (see Table 3).

**Table 3.** Results of Professional Experiences with Mandatory Reporting of Intimate Partner Violence Being Considered Relevant and Choosing Not to Report Regarding a Victim and a Perpetrator Reported in *n* and Valid Percentages.

Frequencies of Cases Where MR-IPV Was Considered Relevant	Victim, *n* (%)	Perpetrator, *n* (%)
0	118 (32.4)	200 (56)
1–5	133 (36.5)	101 (28.3)
6–10	31 (8.5)	14 (3.9)
11–20	27 (7.4)	16 (4.5)
21–30	16 (4.4)	7 (2)
31–40	13 (3.6)	9 (2.5)
41–50	7 (1.9)	3 (0.8)
51–60	4 (1.1)	2 (0.6)
61–70	5 (1.4)	2 (0.6)
71–80	1 (0.3)	0
81–90	2 (0.5)	0
91–100	1 (0.3)	1 (0.3)
100+	6 (1.6)	2 (0.6)
Total, *N*	364	357
Frequencies of choosing not to report		
During the last 12 months		
No	304 (83.5)	347 (97.2)
Yes	60 (16.5)	10 (2.8)
Total, *N*	364	357
Throughout career		
No	256 (71.1)	320 (92)
Yes	104 (28.9)	28 (8)
Total, *N*	360	348

*Note.* Total sample size = 374. MR-IPV = mandatory reporting of intimate partner violence.

Almost half of participants had never reported IPV under MR without or against their victim and perpetrators’ consent or wish, and 40% of participants had never done this with consent (see Table 4). The remaining participants had done this various number of times from 1 to above 10 with and without consent, and some indicated this was not relevant for them. When asked if the participants had ever experienced the following incidents in their professional practice the majority responded no: ever let a victim or perpetrator go home to persistent IPV; ever overridden a victim or perpetrator’s assessment of such a situation; the use of MR was in some way documented within their system (see Table 5).

**Table 4.** Results of Professional Experiences with Reporting With and Without Consent Under Mandatory Reporting Reported in *n* and Valid Percentages.

Frequencies of Ever Reported Under MR-IPV	Without Consent, *n* (%)	With Consent, *n* (%)
0	168 (45.9)	148 (40.5)
1	43 (11.7)	32 (8.8)
2–4	61 (16.7)	55 (15.1)
5–10	30 (8.2)	39 (10.7)
10+	20 (5.5)	41 (11.2)
Not relevant	44 (12)	50 (13.7)
Total, *N*	366	365

*Note.* Total sample size = 374. MR-IPV = mandatory reporting of intimate partner violence.

**Table 5.** Results of Professional Experiences with Different Incidents Related to Evaluation of MR-IPV Reported in *n* and Valid Percentages.

Item	No, *n* (%)	Yes, *n* (%)	Total, *N*
Have you ever let a victim or perpetrator go home to persistent IPV?	239 (67.1)	117 (32.9)	356
Have you ever overridden a victim or perpetrator’s assessment of such a situation?	210 (60.2)	139 (39.8)	349
Have you ever experienced relevant information about the risk of violence not being conveyed, and a victim or perpetrator or their partner subsequently being murdered	348 (97.5)	9 (2.5)	357
Was the use of MR in some way documented within your system?	205 (61.9)	126 (38.1)	331

*Note.* Total sample size = 374. MR-IPV = mandatory reporting of intimate partner violence.

#### Attitudes Toward MR-IPV

As shown in Table 1, 17 statements were presented to the participants to investigate their attitudes toward MR-IPV. Most participants, regardless of profession, were supportive toward MR-IPV. Some statements reached a higher degree of agreement than others (see Table 1). When responses were skewed toward agreement a higher percentage of participants *completely* agreed than the percentage of participants who *completely* disagreed when the responses were skewed toward disagreement. The statement that seemed to reflect the most disagreement across participants (i.e., responses were even across options) concerned whether everyone should have the right to have a doctor or therapist who has *absolute* confidentiality.

### Associations Between Attitudes Toward MR-IPV and Professional Experiences with IPV and MR-IPV

Several predictor variables were significantly associated in regression analyses with the attitude factor score “Skepticism to MR-IPV” used as the outcome (see Table 6 for details). For instance, work experience with IPV (measured by number of cases), both within the last 12 months and throughout career had inverse associations with skepticism to MR-IPV, regardless of the severity of violence or if the cases involved victims or perpetrators. The only variable that was not significant was cases with victims of severe physical injury within the last 12 months. Cases with perpetrators had an overall larger effect size than with victims, meaning that the more cases that participants had with perpetrators overall, the less skeptical they were toward MR-IPV compared to the effect of the experience of cases with victims. This was not tested statistically. Professional experience with IPH was not significant. Having more IPV cases where MR was considered relevant was associated with less skepticism toward MR-IPV, but only if the cases included victims of IPV. There were no significant associations between choosing not to report IPV under MR because the victim or perpetrator requested participants to and being skeptical of MR-IPV.

**Table 6.** Univariate Linear Regression Using “Skepticism to MR-IPV” as Outcome Variable and Number of Cases with IPV, Intimate Partner Homicide and Mandatory Reporting of Intimate Partner Violence as Independent Variables.

Variable	Victims			Perpetrators		
	*R* ^2^	*B*	*p-*Value	*R* ^2^	*B*	*p-*Value
Cases the last 12 months						
IPV	.022	−0.039	**.016** *	.033	−0.094	**.003** ^†^
Severe IPV	.016	−0.055	**.044** ^‡^	.026	−0.125	**.009** ^‡^
Severe physical injury	.009	−0.058	.121^‡^	.031	−0.151	**.004** ^‡^
Cases throughout career						
IPV	.027	−0.031	**.008** **	.041	−0.043	<.001**
Severe IPV	.031	−0.039	**.005** ^‡^	.022	−0.040	**.017** ^††^
Severe physical injury	.022	−0.040	.017**	.024	−0.050	**.012** ^‡^
Ever experienced intimate partner homicide	.004	0.177	.321*	.000	−0.007	.969^‡^
Cases with MR	.040	−0.067	**.001** ^††^	.014	−0.062	.057^‡‡^
Ever not reported because help-seeker asked them not to						
Within the last 12 months	.005	0.151^†^	.279	.000	−0.033	.912***
Throughout career	.010	0.182^†††^	.107	.000	0.041	.825^‡‡‡^

*Note.* Total *N* for the analyses: *264; †260; ‡261; **263; ††262; ‡‡255; ***256; †††258; ‡‡‡250. MR-IPV = mandatory reporting of intimate partner violence.

The longer participants had worked in their current position the less skeptical they were (see Table 7). Regarding awareness of MR-IPV, knowing the law in general and applied to their field, both responding yes and no (using dummy variables in linear regression) were significantly associated with skepticism toward MR-IPV. Participants who responded yes to being aware of the law of MR were less skeptical of MR-IPV, and responding no increased skepticism of MR. Additionally, the variables of having reported with consent and without consent from the victim or perpetrator were significantly predictive of being less skeptical of MR-IPV. However, using dummy variables, only reporting a certain number of times emerged as significant. Reporting 2 to 4 times and more than 10 times was significant for both reporting with and without consent, and reporting 5 to 10 times with consent was also significantly associated with being less skeptical of MR-IPV. The result of the MR report was not significant.

**Table 7.** Univariate Linear Regression Using “Skepticism to MR-IPV” as Outcome Variable and Professional Contextual Variables as Independent Variables.

Independent Variable	*R* ^2^	*B*	β	*p*-Value
Years in position*	.022	−0.015	−.148	**.022**
Do you know the law of MR^†^	.087			**<.001**
To some degree (reference)				
Yes		−0.532	−.189	**.002**
No		0.341	.190	**.002**
Do you know the law of MR in your field^†^	.100			**<.001**
To some degree(reference)				
Yes		−0.572	−.174	**.005**
No		0.367	.221	**<.001**
Reported without consent^‡^	.084			**<.001**
Never (reference)				
Once		−0.212	−.084	.184
2–4 times		−0.419	−.195	**.002**
5–10 times		−0.243	−.085	.174
More than 10 times		−0.826	−.246	**<.001**
Not relevant		0.073	.022	.178
Reported with consent^†^	.081			**<.001**
Never (reference)				
Once		0.082	.031	.630
2–4 times		−0.375	−.163	**.012**
5–10 times		−0.417	−.160	**.012**
More than 10 times		−0.548	−.220	**<.001**
Not relevant		0.082	.027	.667
Result of reporting**	.032			.187
Received (reference)				
Declined		−0.537	−.105	.202
Did not know what to do		−0.164	−.042	.610
Never (reference)		−0.295	−.158	.058
Variable with response options and reference category				
Profession^††^	.109			**<.001**
Emergency department (reference)				
Police		−0.770	−.215	**<.001**
Child welfare workers		−0.638	−.244	**<.001**
Shelter worker		−0.524	−.278	**<.001**
Anger management		−0.567	−.276	**<.001**
Alternative to violence		−0.797	−.342	**<.001**

*Note*. Total *N* for the analyses: *238; †262; ‡261; **149; ††265. MR-IPV = mandatory reporting of intimate partner violence.

### Group Differences Between SPs Regarding Attitudes Toward MR-IPV

Also shown in Table 7, amongst types of professions, only participants from the emergency rooms/sexual assault centers were significantly different than the other professions. They were more skeptical of MR-IPV. However, the longer the participants had worked in their current position, the less skeptical they were toward MR-IPV (see Table 7).

### Multivariate Associations Between Professional Experiences with IPV and MR-IPV and Attitudes Toward MR-IPV

We included, as predictor variables, variables that were significant in univariate analyses (the number of cases with IPV, severe IPV, and severe physical injury, as well as the number of cases where MR was considered relevant, for both victims and perpetrators, during the last 12 months and throughout career). The criterion variable, as in the univariate analyses, was skepticism to MR-IPV. Although not significant in the univariate analysis, the number of cases with victims of severe physical injury within the last 12 months was included in the multivariate analyses, to confirm the lack of significance after adjusting for other variables. In addition, professional experience with violence regardless of severity, time of case, and victim/perpetrator is theoretically important and relatively absent in the literature on associations between experience and attitudes. Additionally, as each main variable was adjusted separately, double-checking this variable would have no impact on the results of the other main variables.

After adjustment, most variables with the number of cases of violence remained significant (see Table 8). The only non-significant variables were the number of cases with victims of severe physical injury during the last 12 months (confirming the univariate analysis), and the number of cases with victims of severe physical injury throughout careers. Notably, all significant variables had a negative effect size (indicating less skepticism toward MR-IPV), except for the number of cases with victims of IPV during the last 12 months. Again, cases with perpetrators of any type of violence had a stronger effect size overall relative to the same type of IPV and time frame for victims.

**Table 8.** Multivariate Linear Regression Using “Skepticism to MR-IPV” as Outcome Variable and Experience with Intimate Partner Violence and Mandatory Reporting of Intimate Partner Violence.

Variable	Victims				Perpetrators			
	*R* ^2^	*B*	β	*p-*Value	*R* ^2^	*B*	β	*p-*Value
Cases the last 12 months								
IPV	.277	0.055	.205	**.030** *	.283	−0.109	−.222	**<.001** ^†^
Severe IPV	.204	−0.059	−.139	**.036** ^‡^	.279	−0.151	−.205	.002**
Severe physical injury	.277	−0.038	−.064	.331*	.283	−0.151	−.190	.004*
Cases throughout career								
IPV	.288	−0.029	−.153	**.020** **	.273	−0.045	−.224	<.001*
Severe IPV	.282	−0.038	−.169	**.010** ^‡^	.273	−0.046	−.178	.007**
Severe physical injury	.277	−0.030	−.111	.092*	.277	−0.045	−.150	**.024** ^‡^
Cases with MR	.277	−0.064	−194	**.003** **	.272	−0.083	−.165	**.013** ^††^

*Note.* Each variable adjusted for: profession; years in profession; knowledge of MR; knowledge of MR within field; reported under MR with consent; reported under MR without consent. Total *N* for the analyses: *231; †227; ‡229; **230; ††225. MR-IPV = mandatory reporting of intimate partner violence.

## Discussion

### Main Findings

The aim of this study was to investigate attitudes toward and professional experiences with MR-IPV amongst SPs. In addition, we evaluated to what extent SPs’ attitudes toward MR-IPV were predicted by professional experience with IPV and MR-IPV, and if there were any group differences amongst types of SPs. These findings are the first to demonstrate statistical associations between professional experience with IPV and MR-IPV and attitudes toward MR-IPV amongst SPs who work with perpetrators and victims of IPV.

### Professional Experiences with and Attitudes Toward MR-IPV Among SPs

In accordance with previous studies, we found that SPs had various degrees of professional experience with MR-IPV (Allert et al., 1997; McDowell et al., 1994; Tilden et al., 1994; Smith et al., 2008). Slightly under half had never reported IPV throughout their career—with or without consent from a victim or perpetrator. Approximately 40% had reported IPV under MR, and roughly 10% considered MR not relevant for them. However, the prevalence of having professional experience with MR-IPV was higher in our study than in previous studies (Allert et al., 1997; McDowell et al., 1994; Smith et al., 2008; Tilden et al., 1994). In contrast to previous studies (Rodriguez et al., 1999; Tilden et al., 1994), participants generally agreed with statements that were supportive of MR-IPV and generally disagreed with statements that were opposed to MR-IPV. Agreements with supportive statements appeared, on a purely descriptive level, to be stronger than agreements to opposing statements.

### Associations Between Attitudes Toward MR-IPV and Professional Experiences with MR-IPV, and IPV

When analyzing our predictor variables with “skepticism to MR-IPV” as the outcome variable, we found that the more IPV victims and perpetrators that participants had worked with, the less skeptical they were of MR-IPV. This finding was consistent regardless of the severity of violence, the timing of the case (last 12 months or throughout the career), or whether the case involved a victim or a perpetrator of IPV. The same result was observed for the number of cases where MR-IPV was considered relevant. These findings remained in adjusted multivariate models. To our knowledge, this is the only study investigating the associations between professional experience with IPV and MR-IPV and attitudes to MR-IPV. Theoretically, there are some possible explanations that can help us better understand the significance of experience.

Festinger’s (1957) theory of cognitive dissonance suggests that individuals change their attitudes in the face of behavior-attitude inconsistency. The results of the current study indicated that less skeptical attitudes to MR-IPV were associated with more professional experience with IPV cases in general and cases where MR-IPV would be considered relevant in particular. If we follow the framework of cognitive dissonance, it might suggest that their experience pushes their attitudes away from skepticism to MR-IPV. We cannot say, based on these results, that SPs are supportive or opposing MR-IPV because of their experiences, just that increase in experience is associated with being less skeptical. As such, it might suggest that SPs perceive nuances of positive and negative aspects of MR-IPV, as opposed to a dichotomized “pro or con” attitude. This is also supported by the theory of ambivalent attitudes, suggesting individuals can hold both negative and positive attitudes toward a target at one time (Harreveld et al., 2015).

### Group Differences Between Types of SPs

Participants from the emergency departments/sexual assault centers were the only SPs significantly more skeptical about MR-IPV than the other SPs. Some previous studies have investigated differences between professions, although only between different types of health personnel. Rodriguez et al. (1999) investigated differences between medical specialties among physicians and found that some specialties were less compliant if a patient would object to reporting. However, this is not directly comparable to our results.

Mgopa et al. (2021) used focus groups (one with employed health personnel and one with health studies students) and found that the health personnel group expressed more positively toward reporting to the police (although they were not mandated to) than the students. This finding concurs with our finding that the longer the participants from emergency rooms had worked in their current position the less skeptical they were regarding MR-IPV. However, because of the qualitative method, professional experience was not measured by cases of IPV or MR-IPV, but rather assigned based on participants being SPs or students.

There might be context-related explanations for the group differences in our study. Confidentiality is highly recognized as a patient right (in Norway and elsewhere), not just among SPs but also the public (Vatnar et al., 2017; WHO, 2014). Although all SP groups included in this study are governed by confidentiality, there might be different perspectives on the primacy of MR-IPV compared to the participants from the emergency department. For instance, the police and child protective services are reliant on other professional groups to put aside confidentiality for the sake of MR for them to follow up both the investigation and for the protection of victims. Hence, it is perhaps not surprising that they would be less skeptical of MR-IPV. Child welfare services also routinely work as mandated reporters with respect to children’s welfare, so they would be more familiar with reporting on behalf of others in a vulnerable position.

The shelter workers, “alternative to violence” and “anger management” therapists, work nearest to IPV victims and perpetrators as their primary line of work. Consequently, they might have more regular professional experiences with MR-IPV and are evaluating and using MR-IPV regularly throughout their work. This might create a more nuanced perspective. Arguably, the SPs in the emergency department are in a more distinct position by working with a larger range of patients than victims and perpetrators of IPV. They might also be less often confronted with the dilemma of confidentiality and MR-IPV than the other groups.

### Strengths and Limitations

The current study adds to the limited research on attitudes toward MR-IPV among SPs. It is the first to explore the association between actual professional experience with IPV and MR-IPV and attitudes toward MR-IPV. The methodology of measuring skepticism toward MR-IPV is a strength, as previous research has not evaluated attitudes toward MR-IPV on a continuum, but rather dichotomously. In addition, the sample included several groups of SPs that had not been explored before and was large compared to previous studies of MR-IPV. Notwithstanding, there are some limitations we would like to acknowledge. Unlike in a longitudinal or an experimental study, we were unable to measure attitude change or causality in the current study. Hence, we cannot infer that professional experience with IPV and MR-IPV and attitudes have a cause-and-effect relationship (i.e., whether experience with MR-IPV has led participants to be less skeptical, or less skepticism has led participants to report more.) We can only conclude that there is a statistical association between the two.

As explained in the methodology, our main outcome variable for this study was a collapsed score created through factor analysis. Unfortunately, due to this method, we had to exclude about 30% of our total sample size. Notably, it did not limit utilizing the type of analyses initially planned. The regression analyses, however, were only done on participants who had responded between “completely disagree” and “completely agree.” Participants who had, at any point, responded “I don’t know” were not included in the model. Responding “I don’t know” is still a valid response, and excluding such responses does lead to a bias toward participants who were, arguably, more conscious about their opinions. Missing responses were excluded in the same manner as “I don’t know” responses. On the other hand, after excluding unusable responses, the final responses were valid data, hence we did not have to impute any responses.

In our multivariate model, we discovered an inconsistency that could not be theoretically explained. In the univariate results, all effect sizes were negative (i.e., less skepticism); however, one significant variable changed directions in the multivariate model. This was the variable covering “cases with victims of IPV within the last 12 months.” Such a finding might result from “overadjustment” (Porta, 2008) because of the complex model (i.e., several adjusting variables). Although overadjustment can be a result of unnecessarily adjusting variables (Schisterman et al., 2009), that was not considered to be the case for this result. There can be several reasons for overadjustment (Lu et al., 2021) but it is difficult to say which one our result falls into. This limits our confidence in this finding.

Additionally, the factor analysis produced below desirable CFI, RMSEA, and alpha, which generate concerns. We acknowledge that there is a common standard for the CFI and RMSEA are above 0.90 and below 0.05, respectively. However, we want to point out that there is research that argues not meeting these criteria does not provide grounds to reject the model (Chen et al., 2008; Marcoulides & Yuan, 2016; van Laar & Braeken, 2021). We are aware that our data was not normally distributed which could contribute to the low CFI (van Laar & Braeken, 2021). More importantly, the factor score used as our dependent variable was not meant to be used as a validated scale, but rather a means to collapse items under a conceptual scale to measure an overarching theme. Consequently, we are confident in our results overall.

### Generalizability

The current study took place in Norway, which has its own national jurisdiction on MR. Hence, we cannot guarantee that these results would generalize to other jurisdictions. Differences in jurisdictions might have an impact on the attitudes among SPs. As a Scandinavian country, Norway has a unique public health and welfare system where the majority of our participants are employed and the IPV victims and perpetrators seek help. Undoubtedly the accessibility to this system and limited financial cost influence the number of individuals who seek help compared to countries where the health system is either more privatized (and depends more on patients’ insurance) or is underdeveloped. In addition, we wanted to include gender inclusivity and allowed participants to respond “other” if they did identify as anything other than male or female, with space to define what this option might be. However, no participant utilized this option.

Our sample is also representative of all counties in the country, which means we would be able to represent areas with fewer inhabitants, local community cultures, as well as cultures within larger cities and communities. Additionally, it would allow for a more diverse sample where participants with foreign nationalities or ethnicities, who are more concentrated in some areas in the country, participate on the same level as native Norwegians and naturalized Norwegian citizens. Simultaneously, we still recognize that Norway is a small country in the global North and may not be generalizable to all other, even Western, or northern-European countries. There might also be cultural differences that cannot be fully accounted for in this study. Notwithstanding, our results are highly relevant internationally as an example of how attitudes toward guidelines and laws can be influenced by different factors and can differ between professions.

### Implications for Future Research

Our findings suggest that although there is general support for MR-IPV among our sample, there are factors that influence the degree of skepticism toward MR-IPV. This is important to consider among SPs. Future research is encouraged within other jurisdictions, in order to test whether our findings are limited to the Norwegian context. Additionally, we encourage exploring other professions that might encounter IPV in other contexts and jurisdictions. We acknowledge there are other factors outside of our study that could influence attitudes and decision-making among professionals, for instance, the influence of authority from administration staff or leaders. This should also be explored. We presented theoretical grounds that might suggest our findings would be generalizable. We also recommend research on whether there are additional factors that might be associated with attitudes toward MR-IPV. Finally, our research will shed light on how attitudes might differ between professions.

## References

[bibr1-08862605241305145] Allert C. S. Chalkley C. M. Whitney J. R. Librett A. (1997). Domestic violence: Efficacy of health provider training in Utah. Prehospital and Disaster Medicine, 12(1), 52–56. 10.1017/s1049023x00037225 10166375

[bibr2-08862605241305145] Antle B. F. Barbee A. P. Yankeelov P. Bledsoe L. K. (2010). A qualitative evaluation of the effects of mandatory reporting of domestic violence on victims and their children. Journal of Family Social Work, 13(1), 56–73. 10.1080/10522150903468065

[bibr3-08862605241305145] Barner J. R. Carney M. M. (2011). Interventions for intimate partner violence: A historical review. Journal of Family Violence, 26(3), 235–244. 10.1007/s10896-011-9359-3

[bibr4-08862605241305145] Bendixen M. (2005). Konflikter og konflikthåndtering blant studenter i parforhold. Norwegian University of Science and Technology. https://www.nb.no/items/URN:NBN:no-nb_digibok_2013082205030

[bibr5-08862605241305145] Chen N. F. Curran P. J. Bollen K. A. Kirby J. Paxton P. (2008). An empirical evaluation of the use of fixed cutoff points in RMSEA test statistic in structural equation models. Sociological Methods & Research, 36(4), 462–494. 10.1177/0049124108314720 19756246 PMC2743032

[bibr6-08862605241305145] Cho R. B. Cha R. K. Yoo R. Y. (2015). Awareness and Attitudes Towards Violence and Abuse among Emergency Nurses. Asian Nursing Research, 9(3), 213–218. 10.1016/j.anr.2015.03.003 26412625

[bibr7-08862605241305145] Costello A. B. Osborne J. (2005). Best practices in exploratory factor analysis: Four recommendations for getting the most from your analysis. Practical Assessment, Research & Evaluation, 10(7), 1–9. 10.7275/jyj1-4868

[bibr8-08862605241305145] Del Rio I. D. Del Valle E. S. G . (2016). Influence of intimate partner violence severity on the Help-Seeking strategies of female victims and the influence of social reactions to violence disclosure on the process of leaving a violent relationship. Journal of Interpersonal Violence, 34(21–22), 4550–4571. 10.1177/0886260516676473 27807209

[bibr9-08862605241305145] Fabrigar L. R. Wegener D. T. MacCallum Robert C Strahan E. J. (1999). Evaluating the use of exploratory factor analysis in psychological research. Psychological Methods, 4(3), 272–299. 10.1037//1082-989X.4.3.272

[bibr10-08862605241305145] Festinger L. (1957). A theory of cognitive dissonance. Stanford University Press.

[bibr11-08862605241305145] Forgas J. Cooper J. Crano W. (Eds.). (2010). The psychology of attitudes and attitude change. Taylor & Francis Group.

[bibr12-08862605241305145] Gaasø O. M. Isaksson Rø K. Bringedal B. Magelssen M. (2019). Legers holdninger til aktiv dødshjelp [Doctor’s attitudes to assisted dying]. Tidsskriftet Den Norske Legeforening.

[bibr13-08862605241305145] Haddock G. Maio G. (Eds.). (2004). Contemporary perspectives on the psychology of attitudes. Taylor & Francis Group.

[bibr14-08862605241305145] Harreveld F. Nohlen H. U. Schneider I. K. (2015). You shall not always get what you want: The consequences of ambivalence toward desires. In Hofmann W. Nordgren L. F. (Eds.), The psychology of desire (pp. 267–285). The Guildford Press.

[bibr15-08862605241305145] Jordan C. E. Pritchard A. J. (2018). Mandatory reporting of domestic violence: What do abuse survivors think and what variables influence those opinions? Journal of Interpersonal Violence, 36(7–8), NP4170–NP4190. 10.1177/0886260518787206 29984619

[bibr16-08862605241305145] Lippy C. Jumarali S. N. Nnawulezi N. Williams E. Burk C. (2019). The impact of mandatory reporting laws on survivors of intimate partner violence: Intersectionality, Help-Seeking and the need for change. Journal of Family Violence, 35(3), 255–267. 10.1007/s10896-019-00103-w

[bibr17-08862605241305145] Lu H. Cole S. R. Platt R. W. Schisterman E. F. (2021). Revisiting overadjustment bias. Epidemiology, 32(5), e22–e23. 10.1097/ede.0000000000001377 34172691

[bibr18-08862605241305145] Lund L. E. (1999). What happens when health practitioners report domestic violence injuries to the police? A study of the law enforcement response to injury reports. Violence and Victims, 14(2), 203–2014. 10.1891/0886-6708.14.2.203 10418772

[bibr19-08862605241305145] Mandated Reporter Training. (2022, October 13). Which states have mandatory domestic violence reporting? https://mandatedreportertraining.com/resources/blog/which-states-have-mandatory-domestic-violence-reporting/

[bibr20-08862605241305145] Marcoulides K. M. Yuan K. (2016). New ways to evaluate goodness of fit: A note on using equivalence testing to assess structural equation models. Structural Equation Modeling, 24(1), 148–153. 10.1080/10705511.2016.1225260

[bibr21-08862605241305145] Mathews B. Walsh K. M. (2014). Mandatory Reporting Laws. In Hayes A. Higgins D. (Eds.), Families, Policy and the Law: Selected Essays on Contemporary Issues for Australia (pp. 131–142). Australian Institute of Family Studies. https://aifs.gov.au/sites/default/files/fp114.pdf

[bibr22-08862605241305145] McDowell J. Kassebaum D. K. Fryer G. E. (1994). Recognizing and reporting domestic violence: A survey of dental practitioners. Special Care in Dentistry, 14(2), 49–53. 10.1111/j.1754-4505.1994.tb01099.x 7871460

[bibr23-08862605241305145] Mgopa L. R. Rosser B. R. S. Ross M. W. Mohammed I. Lukumay G. G. Massae A. F. Mushy S. E. Mwakawanga D. L. Mkonyi E. Trent M. Bonilla Z. E. Wadley J. Leshabari S. (2021). Clinical care of victims of interpersonal violence and rape in Tanzania: a qualitative investigation. International Journal of Women’s Health, Volume 13, 727–741. 10.2147/ijwh.s301804 PMC8318211 34335058

[bibr24-08862605241305145] Panayiotopoulos C. (2011). Mandatory reporting of domestic violence cases in Cyprus; barriers to the effectiveness of mandatory reporting issues for future practice. European Journal of Social Work, 14(3), 379–402. 10.1080/13691457.2010.490936

[bibr25-08862605241305145] Porta M. S. (2008). A dictionary of epidemiology. Oxford University Press.

[bibr26-08862605241305145] Robinson S. R. Ravi K. Schrag R. J. V. (2020). A Systematic review of barriers to formal help seeking for adult survivors of IPV in the United States, 2005–2019. Trauma, Violence & Abuse, 22(5), 1279–1295. 10.1177/1524838020916254 32266870

[bibr27-08862605241305145] Rodríguez M. Sheldon W. R. Rao N. (2002). Abused patient’s attitudes about mandatory reporting of intimate partner abuse injuries to police. Women & Health, 35(2–3), 135–147. 10.1300/j013v35n02_09 12201504

[bibr28-08862605241305145] Rodriguez M. A. Craig A. M. Mooney D. R. Bauer H. M. (1998). Patient attitudes about mandatory reporting of domestic violence. Implications for health care professionals. West J Med, 169(6), 337–341. 9866430 PMC1305400

[bibr29-08862605241305145] Rodriguez M. C. McLoughlin E. Bauer H. M. Paredes V. Grumbach K. (1999). Mandatory reporting of intimate partner violence to police: views of physicians in California. American Journal of Public Health, 89(4), 575–578. 10.2105/ajph.89.4.575 10191807 PMC1508892

[bibr30-08862605241305145] Sawyer S. Parekh V. Williams A. Williams B. (2014). Are Australian paramedics adequately trained and prepared for intimate partner violence? A pilot study. Journal of Forensic and Legal Medicine, 28, 32–35. 10.1016/j.jflm.2014.09.008 25440144

[bibr31-08862605241305145] Schisterman E. F. Cole S. R. Platt R. W. (2009). Overadjustment bias and unnecessary adjustment in epidemiologic studies. Epidemiology, 20(4), 488–495. 10.1097/ede.0b013e3181a819a1 19525685 PMC2744485

[bibr32-08862605241305145] Smith J. Rainey S. L. Smith K. R. Alamares C. Grogg D. (2008). Barriers to the mandatory reporting of domestic violence encountered by nursing SPs. Journal of Trauma Nursing, 15(1), 9–11. 10.1097/01.jtn.0000315782.20213.72 18467941

[bibr33-08862605241305145] Stöckl H. Devries K. Rotstein A. Abrahams N. Campbell J. C. Watts C. Moreno C. (2013). The global prevalence of intimate partner homicide: A systematic review. The Lancet, 382(9895), 859–865. 10.1016/s0140-6736(13)61030-2 23791474

[bibr34-08862605241305145] Straffeloven. (2005). Lov om straff [The Norwegian Penal code]. LOV-2005-05-20-28.

[bibr35-08862605241305145] Straus M. A. Hamby S. L. Boney-McCoy S. Sugarman D. B. (1996). The Revised Conflict Tactics Scales (CTS2). Journal of Family Issues, 17(3), 283–316. 10.1177/019251396017003001

[bibr36-08862605241305145] Tabachnick B. G. Fidell L. S. (2001). Using multivariate statistics. Allyn & Bacon.

[bibr37-08862605241305145] Tilden V. P. Schmidt T. A. Limandri B. J. Chiodo G. T. Garland M. Loveless P. (1994). Factors that influence clinicians’ assessment and management of family violence. American Journal of Public Health, 84(4), 628–633. 10.2105/ajph.84.4.628 8154568 PMC1614797

[bibr38-08862605241305145] United Nations Office on Drugs and Crime. (2022). Global report on trafficking in persons (E.23.IV.1). United Nations Publications.

[bibr39-08862605241305145] Van Dam B. Van Der Sanden W. Bruers J. (2015). Recognizing and reporting domestic violence: Attitudes, experiences and behavior of Dutch dentists. BMC Oral Health, 15, 159. 10.1186/s12903-015-0141-4 26667115 PMC4678711

[bibr40-08862605241305145] van Laar S. Braeken J . (2021). Understanding the comparative fit index: It’s all about the base! Practical Assessment, Research, and Evaluation, 26, 26. 10.7275/23663996

[bibr41-08862605241305145] Vatnar S. K. B. Friestad C. Bjørkly S. (2017). Intimate partner homicide in Norway 1990–2012: Identifying risk factors through structured risk assessment, court documents, and interviews with bereaved. Psychology of Violence, 7(3), 395–405. 10.1037/vio0000100

[bibr42-08862605241305145] Vatnar S. K. B. Leer-Salvesen K. Bjørkly S. (2021). Mandatory reporting of intimate partner violence: A mixed methods systematic review. Trauma, Violence, & Abuse, 22(4), 635–655. 10.1177/1524838019869102 31446848

[bibr43-08862605241305145] Walker R. M. (2017). Mandatory reporting of Intimate partner violence: an ethical dilemma for forensic nurses. Journal of Forensic Nursing, 13(3), 143–146. 10.1097/jfn.0000000000000159 28700382

[bibr44-08862605241305145] World Health Organization (WHO). (2012). Understanding and addressing violence against women (WHO/RHR/12.43). World Health Organization. https://www.who.int/publications/i/item/WHO-RHR-12.43

[bibr45-08862605241305145] World Health Organization (WHO) (2013). Responding to intimate partner violence and sexual violence against women WHO clinical and policy guidelines (978 92 4 154859 5). World Health Organization. 24354041

[bibr46-08862605241305145] World Health Organization (WHO) (2014). Global status report on violence prevention 2014. World Health Organization. https://www.who.int/publications/i/item/9789241564793

[bibr47-08862605241305145] World Health Organization (WHO) (2017). Responding to children and adolescents who have been sexually abused, WHO clinical guidelines. (978 92 4 155014 7). https://iris.who.int/bitstream/handle/10665/259270/9789241550147-eng.pdf?sequence=1 29630189

